# Guts, Germs, and Iron: A Systematic Review on Iron Supplementation, Iron Fortification, and Diarrhea in Children Aged 4–59 Months

**DOI:** 10.1093/cdn/nzz005

**Published:** 2019-01-15

**Authors:** Adnaan Ghanchi, Philip T James, Carla Cerami

**Affiliations:** 1 Faculty of Epidemiology and Population Health, London School of Hygiene & Tropical Medicine, Keppel Street, London, United Kingdom; 2 Nutrition Theme, Medical Research Council Unit The Gambia at the London School of Hygiene & Tropical Medicine, Atlantic Boulevard, Fajara, Banjul, The Gambia

**Keywords:** iron, ferrous sulfate, iron deficiency anemia, diarrhea, anemia, multiple micronutrient powder

## Abstract

**Background:**

The impact of iron supplements and iron fortification on diarrhea in children is controversial, with some studies reporting an increase and others reporting no effect.

**Objective:**

The aim of the study was systematically assess the published literature on oral iron supplementation and fortification to evaluate its impact on diarrhea incidence among children aged 4–59 mo.

**Methods:**

Randomized controlled trials of oral iron supplementation or iron fortification that reported diarrheal outcomes in children aged 4–59 mo were identified from a systematic search of 5 databases.

**Results:**

Of the 906 records identified, 19 studies were found to fit the inclusion criteria for this systematic review. However, variable case definitions for diarrhea made meta-analysis impossible. Of the 19 studies, 7 (37%) studies showed a significant increase, either in overall diarrhea incidence or within a specific subgroup of the population, between iron-supplemented and control groups. Subgroups included children who were iron-replete and children undergoing their first month of iron intervention. Two studies reported an increase in bloody diarrhea. The remaining 12 (63%) studies showed no difference between iron-supplemented and control groups.

**Conclusions:**

Studies on iron supplementation and fortification use divergent case definitions for diarrhea. A number of studies (37%) showed an increase in overall diarrhea incidence or within a specific subgroup of the population, between iron-supplemented and control groups, but the majority (63%) did not. In addition, there was no clear relation between diarrhea and type of intervention or amount of iron administered observed. In future studies, we recommend that diarrhea be clearly defined and consistently recorded as a secondary outcome. Antibiotic status of participants receiving iron should also be collected to help assess possible drug interactions resulting in a “red stool effect.” Finally, further microbiome research is required to better understand the effects of oral iron on specific bacterial species in the colon.

## Introduction

Iron is required for many essential metabolic processes (1). Pathogens and humans require iron and have developed complex ways to acquire, transport, and store it (2). Bacteria have developed multiple mechanisms for chelating iron and heme directly and for acquiring iron attached to various human iron chaperone molecules (3). In turn, humans tightly regulate free iron at a molar concentration of less than 10^24^, and bind it with proteins such as ferritin, transferrin, and lactoferrin (4).

Iron deficiency anemia occurs when both intake and total body iron are insufficient to meet the needs of erythropoiesis. A 2011 WHO report estimated a prevalence of 43% of anemia

worldwide (5), with over half of cases attributable to iron deficiency (6). Infants aged 0–5 y, pregnant women, and women of childbearing age are at highest risk (7). Relative iron requirements for children are higher than adults because of the nutritional demands of accelerated growth (8). Among its many uses, iron is essential for brain growth: it is necessary for myelination of oligodendrocytes (9) as well as the production of the key neurotransmitters serotonin (10) and dopamine (11).

In low-income countries, there is concern that untargeted iron supplementation can predispose children to certain infections, including malaria, diarrhea, and respiratory infections. One previous systematic review published in 2002 analyzed 28 randomized controlled trials (no age limits on participants) for the effect of both iron supplementation (oral and parenteral) and fortification on a number of infectious disease outcomes (12). In the analysis, subjects receiving iron had a higher risk of only 1 complication, diarrhea (at an 11% increase).

Iron supplementation and fortification could induce diarrhea by causing intestinal damage through oxidative stress (13–16) or by initiating bacterial dysibiosis and gut inflammation (17–20).

This review focuses specifically on children from the ages of 4 mo to 5 y, a population group that is concurrently at the highest risk of diarrhea and most likely to benefit from iron intervention (8, 21). The primary objective is to systematically assess the published literature on oral iron supplementation and fortification to evaluate its impact on diarrhea incidence among children aged 4–59 mo. Secondary objectives include establishing whether any specific population subgroups are at increased risk of diarrhea and discussing possible potential policy implications based on the results found.

## Methods

### Search strategy

This systematic review adheres to the Preferred Reporting Items for Systematic Review and Meta-Analysis (PRISMA) 2009 Checklist (**Supplemental Table 1**) (22). We published the protocol for this study on 19 May 2017 (CRD42017067297). We conducted a systematic search across 5 different databases; Medline (1946 to July 2017), EMBASE (1974 to 2017 week 31), Global Health (1910 to 2017), Web of Science, and Cochrane Central Register of Controlled Trials. The last search was conducted on 31 July 2017. The search strategy consisted of 4 main concepts: “children,” iron,” “supplementation/fortification,” and “diarrhea” (Supplemental Table 1). Owing to the similarity of Boolean operators, Ovid was used to retrieve searches from 3 databases—Medline, EMBASE, and Global Health—simultaneously. For studies that were indexed by the search but inaccessible, relevant authors were contacted to retrieve full texts. Through this method, 1 further full text was made accessible (23).

### Inclusion and exclusion criteria

We restricted the review to double-blind, randomized controlled trials in humans. Searches were limited to the English language. Inclusion criteria were predefined in the published protocol and are reported in the Cochrane endorsed population intervention comparator outcome format (24): population—children between the ages of 4 mo and 5 y at the initiation of iron intervention; intervention—oral iron supplementation or fortification of any kind, any dose and any duration, including multiple micronutrient supplementation if iron was a principal component; comparator—any placebo or control group of the same population receiving no intervention or an intervention containing negligible amounts of additional iron; outcome—diarrhea or dysentery cases reported as either a primary or secondary outcome in any format. We excluded review articles, case studies, and unpublished trials. Studies that obtained participants with existing cases of diarrhea were excluded because they were unrepresentative of the general population. Nonoral iron supplementation, formulated foods, lipid-nutrient supplementation, meat-based iron supplementation, infant formula milk, fortified breast-milk, and bovine lactoferrin were all excluded. Owing to existing evidence that zinc supplementation reduces risk of diarrhea (25, 26), we excluded trials that combined the iron and zinc supplementation arm, unless there was also an iron-only arm. Owing to frequent inconsistencies in the case definitions for diarrhea, we included all case definitions of diarrhea as described in the studies.

### Analysis

Owing to substantial heterogeneity of reported outcomes, it was not possible to conduct a meta-analysis, and instead a vote-counting method was used. Studies were classified as either increasing risk of diarrhea with iron formulation/supplementation or having no effect using a significance level of *P* < 0.05. We described the overall trend of the studies, with a focus on whether any population subgroups or intervention types were particularly affected by iron supplementation or fortification.

### Risk of bias

All studies progressing to the extraction phase were assessed using the Cochrane risk of bias tool to ensure adequate quality (27). Categories assessed included: selection bias, detection bias, attrition bias, reporting bias, and “other” biases (such as poor case definitions as well as weak methods of outcome detection).

Every study was assessed for each category of bias individually, and a judgment was made to score the bias as “low risk,” “high risk,” or “unclear risk” if information was insufficient. The bias scores in each category were then used to obtain an overall statement of study quality. Studies were initially considered to be “high quality” and were downgraded to “adequate quality” and “low quality” for each additional category containing a high risk of bias. Studies that scored an “unclear” risk in 4 or more categories were also downgraded in quality.

All studies progressing to the final stage of the review underwent full data extraction regardless of risk of bias. Risk of bias data was recorded and assessed using RevMan v5.2 to display quality outcomes both within and between studies.

## Results

### Included studies

**FIGURE 1:**
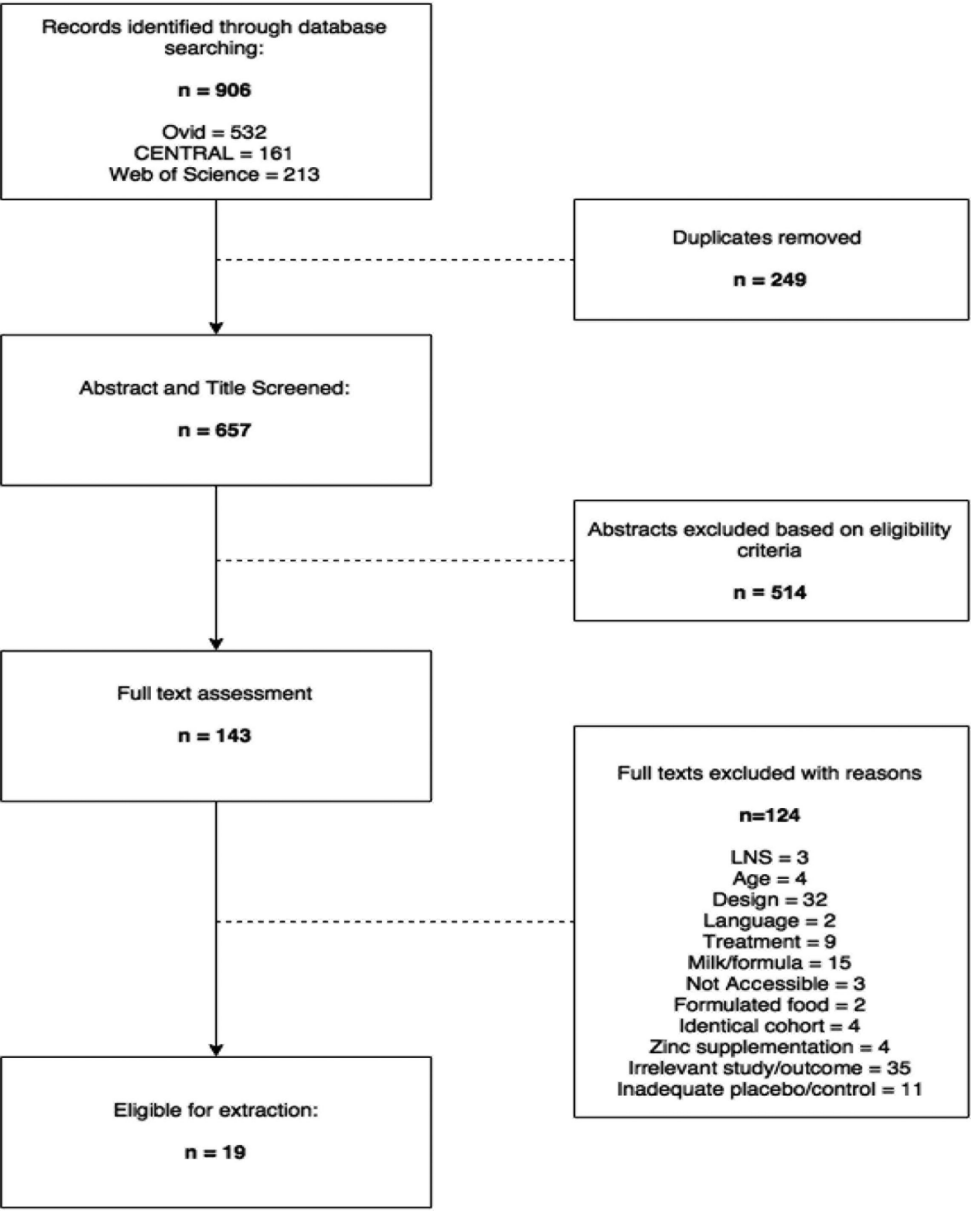
Study selection flow diagram. LNS, lipid-based nutrient supplement.

As detailed in **Figure 1**, a total of 906 records were identified using the predefined search strategy **(**Supplemental Table 1). A total of 249 duplicates were removed, and of the remaining 657 studies, 143 were eligible for full text appraisal. Four potentially relevant texts were deemed inaccessible. Corresponding authors were contacted, and 1 text was successfully retrieved (23). A full list of excluded studies with reasons for exclusion is available in **Supplemental Table 2**.

Nineteen studies progressed to the final stage of review and ranged in publication date from 1991 to 2017. The papers summarized global data; 9/19 studies were from Asia (3 from Bangladesh, 2 from Pakistan, 2 from China, 1 from Cambodia, 1 from India), 4 from Africa (3 from Kenya, 1 from South Africa), 4 from North America (Canada, Honduras, Haiti, Mexico), 1 from South America (Peru), and 1 from Europe (Sweden). One study, undertaken by Dewey et al. (28), included 2 simultaneous cohorts from both Honduras and Sweden. As such, these cohorts have been considered separately in the analyses.

### Study design

All 19/19 studies were randomized controlled trials, with 7 being of a simple design, containing a placebo and iron-intervention group only. The remaining studies (12/19) included multiple intervention arms (**Tables 1** and **2**). Only 2 studies did not randomly assign individual participants: Menon et al. (29) and Soofi et al. (30), who used cluster randomization of villages and food distribution points respectively. The total duration of intervention varied greatly, from 2 to 18 mo. One study, Chen et al. (31), showed a modestly inflated study duration, as the intervention was suspended during weekends and holidays, leading to the reported length of 6 mo being equivalent to 120 supplementation days (4 mo).

**TABLE 1:** Summary table of included studies: fortification^1^

Study	Age: duration	Iron type	Intervention, *n*	Control, *n*	Total, *n*	Other study arms, *n*	Effect size	*P* value	Subgroups of note	Detrimental effect of intervention
Barth-Jaeggi et al*.* (67); 2015; Kenya	6 mo: 12 mo	2.5 mg NaFeEDTA	88	82	170		Proportion: intervention 26%, placebo 29%	*P* > 0.05		No
Chen et al. (31); 2011; China	2–6 y: 6 mo	12 mg NaFeEDTA	71	61	226	94 MMN	Risk ratio: intervention 0.95(0.81–1.15), intervention vs. MMN 0.78 (0.61–0.92)	*P* > 0.05		No
Christofides et al*.* (62); 2005; Canada	4–18 mo: 6 mo	30 mg ferrous fumarate	26	36	62		Risk ratio: 1.09 (0.61–1.97)			No
Giovannini et al. (68); 2006; Cambodia	6 mo: 12 mo	12.5 mg ferrous fumarate	68	68	204	68 MMN	Proportion: intervention 10.3%, placebo 5.9%, MMN 10.3%			No
Jaeggi et al. (17); 2014; Kenya	6 mo: 4 mo	12.5 mg ferrous fumarate	22	24	46	47 ferrous pyrophosphate	Proportion: intervention 27.3%, placebo 8.3%	*P* = 0.092	Another formulation of 2.5 mg iron was assessed, but morbidity data not provided	No
Javaid et al*.* (69); 1991; Pakistan	4.4 mo: 8 mo	4.1–5.1 mg (mean) ferrous fumarate	40	42	129		Episodes per infant: interventions 2.87, placebo 2.49	*P* > 0.05	A separate control group with no cereal or fortification showed a rate of 4.32 episodes per infant	No
Lemaire et al*.* (33); 2011; Bangladesh	12–24 mo: 2 mo	12.5 mg ferrous fumarate	132	126	258		Number of cases: intervention 126, placebo 135	*P* > 0.05	Two separate cohorts used: winter/summer	No
Menon et al*.* (29); 2007; Haiti	9–24 mo: 2 mo	12.5 mg “iron”	254	161	415		Proportion: intervention 58% control 43% (first month only)	*P* < 0.05	Additionally: nonanemic children had a higher prevalence of diarrhea than anemic children in the intervention group during the first month *P* < 0.05; an increase in diarrhea morbidity from baseline to month 1 was also seen in the fortified nonanemic group *P* < 0.13	Yes
Paganini et al*.* (23); 2017; Kenya	6.5–9.5 mo: 4 mo	12.5 mg “iron,” 2.5 mg NaFeEDTA	52	51	154	52 iron + galacto-oligosacharides	Number of cases in all groups: 74; quote: “no significant group differences in the number of infants treated for diarrhea”	*P* > 0.05		No
Soofi et al*.* (70); 2013; Pakistan	6 mo: 12 mo	12.5 mg ferrous fumarate	746	779	2271	746 MNP + zinc	Incidence rate: intervention 4.16, control 3.73, MNP + zinc 4.32	*P* = 0.12	Increased incidence of bloody diarrhea between 6 and 18 mo in both MNP groups *P* = 0·003; weaker evidence of an increase in severe diarrhea among children receiving MNPs (≥ 6 stools per day) *P* = 0·07	Yes

1 MMN, multiple micronutrient; MNP, micronutrient powder; NaFeEDTA, sodium iron ethylene diamine tetraacetate.

**TABLE 2:** Summary table of included studies: supplementation^1^

Study	Age: duration	Iron type	Intervention, *n*	Control, *n*	Total, *n*	Other study arms, *n*	Effect size	*P* value	Subgroups of note	Detrimental effect of intervention
Abdelrazik et al*.* (38); 2007; India	6 mo: 12 mo	43 mg ferrous gluconate	198	50	348		Proportion: intervention 75.8%, placebo 50%	*P* = 0.03	Of the group that received iron, those with normal ferritin at baseline had higher rates of diarrhea; *P* = 0.04	Yes
Baqui et al*.* (36); 2003; Bangladesh	6 mo: 6 mo	20 mg ferrous sulfate	165	157	476	154 MMN	Adjusted odds: iron 1.01(0.91–1.13), MMN 1.15 (1.02–1.29), control 1.0	*P* < 0.05	A zinc and zinc + iron arm was excluded from extraction; MMN was not well tolerated with a 41% drop out rate; iron alone had little effect on diarrhea incidence, but MMN containing iron had a significant impact	Yes
Chang et al*.* (34); 2010; Bangladesh	6–18 mo: 6 mo	6.25 mg “Iron”	201	201	799	199 iron and zinc, 198 zinc only	Incidence rate: iron 2.7, placebo 2.3, combined iron and zinc 2.0, iron and zinc alternative days 2.1, zinc alone 2.3.	*P* < 0.05	Iron alone significantly increases risk of diarrhea in children; this effect is mitigated by the addition of zinc; giving iron to underweight children had less detrimental effects on diarrhea than those who were of normal weight.	Yes
Chen et al*.* (71); 2013; China	3–6 y: 6 mo	1–2 mg/kg ferrous sulfate	98	104	292	90 vitamin A + iron	Incidence rate: iron 0.4, placebo 0.43, vitamin A + iron 0.28	*P* > 0.05	Significant decrease when iron is combined with vitamin A *P* < 0.05	No
Dewey et al. (28); 2002; Honduras Cohort	4 mo: 3 or 5 mo	1 mg/kg ferrous sulfate	36/40 (3/5 mo)	42	118		Proportion (over whole study duration): intervention 64%/58% placebo 50%		Iron supplementation reduced the risk of diarrhea among infants with Hb < 110 g/L at 4 mo, but led to an increase in diarrhea among infants with Hb > 110 g/L at 4 mo *P* = 0.03; NB: combined cohorts used: Sweden/Honduras; morbidity data specific to 4–6 mo and 6–9 mo reported, but overall morbidity incidence extracted only	Yes
Dewey et al*.* (28); 2002; Swedish Cohort	4 mo: 3 or 5 mo	1 mg/kg ferrous sulfate	30/30 (3/5 mo)	36	96		Proportion (over whole study duration): intervention 27%/30% placebo 14%		Combined data, as above	Yes
Luabeya et al. (32); 2007; South Africa	6 mo: 18 mo	10 mg ferrous fumarate	109	113	335	113 zinc + vitamin A	Number of cases: intervention 89 placebo 98 vitamin A + zinc 92	*P* = 0.484		No
Mitra et al*.* (35); 1997; Bangladesh	29 mo: 15 mo	125 mg ferrous gluconate	118	131	249		Diarrhea episodes per child per year: intervention 2.8 (1.6–4.8), control 2.5 (1.6–5.0) dysentery episodes per child per year: intervention 2.5 (0.9–4.8), control 2.5 (0.9–4.8)	Diarrhea *P* = 0.32, dysentery *P* = 0.84	Dysentery episodes per child per year, under 12 months of age: iron 5.2 (2.4–7.8), control 3.5 (2.1–4.8); *P* = 0.03	Yes
Richard et al*.* (72); 2006; Peru	0–4 y (multiple strata): 7 mo	15 mg ferrous sulfate	60	61	187	66 zinc	Risk ratio: intervention 0.97 (0.78–1.21), iron + zinc 0.89 (0.70–1.12), control 1.0.	*P* = 0.32		No
Rosado and Allen (73); 1997: Mexico	1.5–3 y: 12 mo	20 mg ferrous sulfate	54	56	165	55 iron + zinc	Episodes per year: intervention 76, iron + zinc 46, placebo 62	*P* > 0.05 intervention *P* < 0.05 iron + zinc		No

1 Hb, hemoglobin; MMN, multiple micronutrient.

### Population

Four out of 19 studies recruited their participants in a hospital setting either through routine infant clinics or at birth, 13/19 studies recruited from predefined geographical areas, and 2/19 enrolled nursery attendees. Eleven out of 19 studies selected children under the age of 12 mo, with 9 of these selecting children 6 mo old or older, as they began complementary feeding. Two studies, Luabeya et al. (32) and Lemaire et al. (33), recruited participants with existing comorbidities: HIV infection and moderate acute malnutrition respectively. All other studies detailed specific inclusion and exclusion criteria, excluding participants with congenital malformations or chronic diseases (**Supplemental Table 3**).

### Intervention style

Nine out of 19 studies involved direct supplementation of iron through the use of syrups, tablets, capsules, and solutions. Five studies involved traditional fortification: 2 studies using maize, 1 study using wheat, and 2 studies using a nondescript cereal for fortification. “Point of use fortification,” often interchangeably termed “sprinkles,” “micronutrient powders,” or “at-home fortification,” was used by 5 studies (**Table 3**).

**TABLE 3:** Reported effect of intervention on diarrhea incidence by iron type^1^

Iron type	Increased incidence	No effect
Ferrous sulfate	Dewey et al*.* (28)	Richard et al*.* (72)
Ferrous sulfate	Baqui et al*.* (36)	Chen et al. (2013) (71)
Ferrous sulfate		Rosado and Allen (73)
Ferrous gluconate	Abdelrazik et al*.* (38)	
Ferrous gluconate	Mitra et al*.* (35)^2^	
Ferrous fumarate	Soofi et al*.* (30)^2^	Jaeggi et al*.* (17)
Ferrous fumarate		Luabeya et al*.* (32)
Ferrous fumarate		Lemaire et al*.* (33)
Ferrous fumarate		Christofides et al*.* (62)
Ferrous fumarate		Javaid et al*.* (69)
Ferrous fumarate		Giovannini et al*.* (68)
NaFeEDTA + ferrous fumarate		Paganini et al*.* (23)
NaFeEDTA		Barth-Jaeggi et al*.* (67)
NaFeEDTA		Chen et al*.* (2011) (31)
Nondescript iron	Chang et al*.* (34)	
Nondescript iron	Menon et al*.* (29)^2^	

1 NaFeEDTA, sodium iron ethylene diamine tetraacetate.

2 Significant increase within population subgroup only.

### Intervention type

Two studies, Menon et al. (29) and Chang et al. (34), did not specify the type of iron used. The remaining studies used iron sulfate, fumarate, gluconate, sulfate, or sodium iron ethylene diamine tetraacetate (NaFeEDTA). One study, Paganini et al. (23), used a novel combination of 2 forms of iron: ferrous fumarate and NaFeEDTA (**Table 4**).

**TABLE 4:** Reported effect of intervention on diarrhea incidence by intervention style^1^

Intervention category	Intervention form	Increased incidence	No effect
Fortification	Cereal		Chen et al*.* (2011) (31)
	Cereal		Javaid et al*.* (69)
	Wheat soy blend	Menon et al*.* (29)^1^	
	Maize		Jaeggi et al*.* (17)
	Maize		Barth-Jaeggi et al*.* (67)
Point-of-use fortification	Sprinkles	Soofi et al*.* (30)^1^	Christofides et al*.* (62)
	Sprinkles		Lemaire et al*.* (33)
	Sprinkles		Paganini et al*.* (23)
	Sprinkles		Giovannini et al*.* (68)
Supplementation	Syrup	Abdelrazik et al*.* (38)	Richard et al*.* (72)
	Syrup	Mitra et al*.* (35)^1^	
	Syrup	Dewey et al*.* (28)	
	Tablet/capsule	Baqui et al*.* (36)	Luabeya et al*.* (32)
	Tablet/capsule		Chen et al*.* (2013) (71)
	Dissolvable tablet/solution	Chang et al*.* (34)	Rosado and Allen (73)

1 Significant increase within population subgroup only.

### Intervention frequency

All studies provided participants with a regime of daily iron supplementation, besides Chang et al. (34) who supplemented on alternate days (**Supplemental Table 4**). Four out of 19 studies adjusted their iron dose according to either weight or age, with the remainder using a fixed dose of daily iron. Significant heterogeneity in amount of iron delivered was seen between studies. A full list of detailed ingredients and intervention types for each intervention is provided in **Supplemental Table 5**.

### Outcome

Five out of 19 studies did not provide a specific case definition for the diagnosis of diarrhea, with only 10 of 19 studies using the standard definition of “three or more watery stools within a 24 hour period.” In all studies, cases were reported by infants’ mothers to a fieldworker or study personnel via informal interviews or written questionnaires. One study, Mitra et al. (35), deviated from this method by additionally including physician's records when measuring diarrhea incidence (Supplemental Table 3).

### Effect of intervention

Twelve out of 19 (63%) studies showed no significant difference in diarrheal outcomes between intervention and placebo groups. Four out of 19 (21%) studies showed a significant difference in diarrhea incidence between groups, with all reporting an increase in morbidity. Reported outcome formats varied greatly with a variety of rates, ratios, proportions, and raw numbers all being presented. A further 3 out of 19 (16%) studies showed a higher rate of diarrhea in specific subgroups only. Mitra et al. (35) demonstrated strong evidence of an increased rate of dysentery (defined as mucus or blood-containing stool) in children under 1 year of age. Menon et al. (29) reported a significant increase in diarrheal incidence in iron-replete infants during the initial month of supplementation. Soofi et al. (30) also presented a strongly significant increase in bloody diarrhea within those taking iron-containing micronutrient powders.

Although no formal statistical analyses were conducted, no clear relation between style of supplementation and diarrheal incidence was evident. Baqui et al. (36) showed no effect of 20 mg ferrous sulfate supplementation alone but an increased incidence in diarrheal morbidity when iron was delivered as a multiple micronutrient formation. None of the 3 studies that used NaFeEDTA led to increases in diarrheal incidence. There was also no clear relation between volume of iron administered and diarrheal outcomes.

### Risk of bias

**FIGURE 2:**
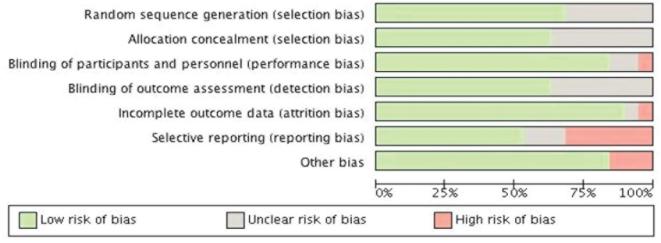
Cochrane risk of bias graph for included studies.

The risk of bias assessment was determined on all 19 studies (**Table 5** and **Figure 2**). The overall risk of bias was low, with 9/19 (47%) studies considered “high” quality, a further 8/19 (42%) being of “adequate,” and just 2/19 (11%) being considered “low” quality. Between studies, the most common risk of bias was that of reporting bias with 6/19 (31%) studies scoring inadequately in this area. For the majority of papers, diarrhea was not a primary outcome and often only added as an aside to the original results if deemed noteworthy.

**TABLE 5:**
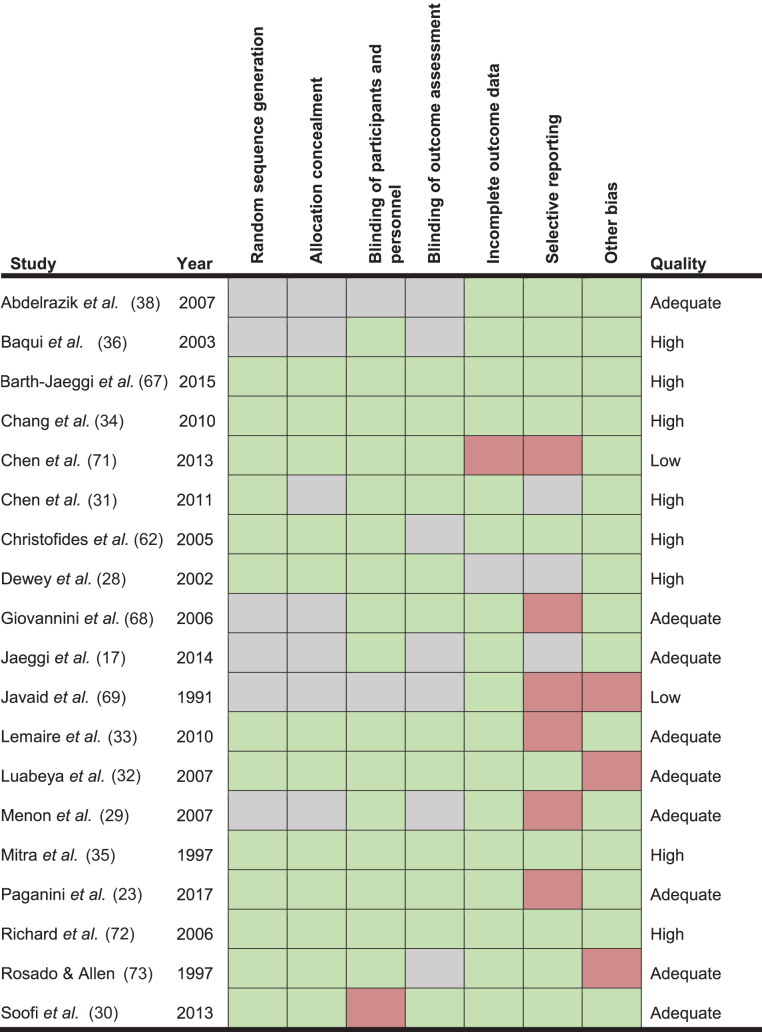
Risk of bias assessment for included studies^1^

All studies (19/19) reported random sequence generation, but 6/19 did not disclose their exact methods of randomization. Performance bias was adequately addressed in 16/19 papers, with only Soofi et al. (30) being downgraded to “high risk” because of a lack of use of an adequate placebo. Papers with the lowest risk of bias in this category, such as Jaeggi et al. (17), used triangle taste testing to ensure participants could not discriminate between iron compounds and placebo. Blinding of outcome assessors was described in 12/19 studies and unreported in the remainder. Studies not reporting this bias category tended to be older, predating the 2010 Consolidated Standards of Reporting Trials (CONSORT) criteria (37). Seventeen out of 19 studies provided attrition data, usually via an annotated flow diagram accounting for loss to follow-up for each individual.

Six out of 19 studies were categorized as being at high risk of reporting bias; this was mainly due to the incomplete presentation of both absolute and relative values for diarrhea incidence. In many cases, this was because diarrhea was not an intended primary outcome. One study by Lemaire et al. (33) was suspected of reporting bias due to the presentation of a composite outcome consisting of dysentery, diarrhea, and lower respiratory tract infections as a single figure. In order to account for this, supplementary data for this study were located to retrieve outcome-specific results. When considering “other” sources of bias, 3/19 studies were penalized for having weak or nondescript case definitions. Supporting statements for each risk of bias judgment are provided in **Supplemental Table 6**.

## Discussion

In summary, of the 19 studies extracted, 12 showed no effect of iron on diarrheal incidence, 4 showed a significant increase, and a further 3 showed an increase within a specific subpopulation.

### Iron and pathogen-induced diarrhea

Iron supplement/fortificant-induced diarrhea could be due to 2 candidate mechanisms: first, through the production of reactive oxygen species and second through bacterial dysibiosis.

Iron itself has the potential to produce copious reactive oxygen species within the intestinal tract through both the Haber–Weiss and Fenton reactions (14). This has the unintended side-effect of causing intestinal damage through oxidative stress, thus precipitating lipid peroxidation and inflammatory diarrhea (13). This mechanism has been demonstrated in in vitro studies with enterocyte-like cells exhibiting a degradation in epithelial integrity after iron exposure (15, 16).

Two recent randomized controlled trials have shown that iron intervention can alter the gut microbiome (17, 18). Specifically, both studies observed a trend toward increase in *E.coli* as well as a concurrent decrease in Lactobacillaceae (19). Both studies also showed a significant increase calprotectin within the intervention group, a biomarker for gut inflammation.

Because there are multiple biologically plausible mechanisms by which oral iron supplementation could cause diarrhea and conflicting clinical data, we attempted to perform an analysis of the current literature to assess the possibility that a causal relation exists.

### Diarrhea in the iron-replete

Four cohorts, Abdelrazik et al. (38), Menon et al. (29), Dewey et al. Sweden/Honduras (28), extracted in this review displayed a higher incidence of diarrhea specifically in children who were iron-replete as defined by the study. This review's findings, that iron-replete individuals may be more susceptible to iron-induced diarrhea, support current WHO guidelines that recommend the use of iron fortificant or supplements only in areas that have an anemia prevalence of 40% and 20% respectively (39, 40).

### Bloody diarrhea

Two studies, by Soofi et al. (30) and Mitra et al. (35), showed a significantly increased incidence of acute bloody diarrhea within those who received iron interventions. Acute bloody diarrhea, commonly referred to as dysentery, is a symptom commonly associated with toxin-producing bacteria such as *Shigella*, *E.coli*, *Salmonella*, or *Campylobacter* (41). The results presented by the Pakistan study were alarming enough to prompt a correspondence in *The Lancet* in 2013 with Tobe-Gai et al., who called for an “urgent need… (for) robust evidence on age-specific doses” of micronutrient powder (42). Although it is tempting to attribute the results to iron administration, 1 further possibility is that of antibiotic interactions. Unlike other cohorts, such as those in Jaeggi et al. (17) and Paganini et al. (23), the Pakistan study included participants receiving antibiotic treatments at baseline. A candidate antibiotic that may be accountable for the significant difference in dysenteric outcomes is Cefdinir (43). Cefdinir is a third-generation cephalosporin often used in pediatric populations for the treatment of penicillin-resistant infections such as otitis media, sinusitis, and pharyngitis (44–46) These infections are extremely common in infants and also have an increasingly high resistance to first-line antibiotics with recent reports estimating between 30% and 70% resistance (47). One side-effect of Cefdinir that is becoming increasingly well documented is its ability to cause the formation of red stools, especially when coadministered with iron (48). Based on case reports, the volume of iron needed to form these red-iron complexes is relatively low (49). One small randomized controlled trial described the frequency of stool discoloration from Cefdinir to be as high as 10%, with a concurrent significant increase in diarrhea at higher doses (50). This relatively common Cefdinir side-effect may falsely promote an apparent association between iron administration and bloody diarrhea.

It is plausible that, especially in a sample of almost 3000 infants, a plethora of antibiotics were prescribed, dependent on availability and prescribing patterns of the region. It could be argued that both the Pakistan cohort and the Mitra et al. (35) Bangladesh cohort did not show an increase in bloody diarrhea for all children, but only those younger than 18 mo. If cephalosporin administration was accountable for this relation, differential prescribing between age groups would have to be demonstrated. Alternatively the association could be explained by the epidemiology of otitis media itself, which has a peak incidence during the first year of life, specifically 6–18 mo (51). During this period, we would expect the prescription of cephalosporins to be most frequent and thus the incidence of reported bloody diarrhea to be higher, as is the case with both studies. Although unlikely, if antibiotic prescription were liable for some of the results observed, the ramifications of these findings would be significant.

### Risk of diarrhea by type of intervention

Five out of 9 iron supplementation studies showed a significant increase in the diarrhea. There is little consensus on which iron type should be used. However, ferrous fumarate provides the most iron per gram, ferrous sulfate is the cheapest, and ferrous gluconate is known for its minimal side-effect profile (52). Fourteen studies utilized conventional iron salts as a form of iron intervention. These include ferrous fumarate, ferrous sulfate, and ferrous gluconate, in order of decreasing bioavailability (53).

Three studies utilized NaFeEDTA, with all of these studies showing no effect on diarrheal morbidity. The benefits of NaFeEDTA are 3-fold. First, within the lumen of the intestine, the unconventional manner in which the EDTA complex binds iron may sequester iron from iron-dependent pathogens, thus withholding iron desperately needed for survival (17). Second, it is well established through in vitro experimentation that EDTA itself exhibits antimicrobial properties and is commonly used to prevent the formation of biofilm. Recent studies have pertinently shown that EDTA can induce the deterioration of both *E. coli* and *Salmonellaenterica* cell membranes (54, 55). Finally, when used as a fortificant, the EDTA component also protects iron from the inhibitory effect of phytates and polyphenols (56). Moreover, NaFeEDTA has been reported to be absorbed 2–4 times more efficiently than ferrous sulfate, the compound once considered the benchmark of iron bioavailability (57, 58). Its use has been recently endorsed by the WHO/FAO Expert Committee on Food Additives and is recommended for use specifically with corn and condiments (56). Despite its inherent benefits, NaFeEDTA is expensive, its effective cost per milligram reported to be 16 times that of ferrous sulfate alone, making it less viable for resource-poor programs (59).

Fortification is often considered as a safer alternative to supplementation because of its smaller dose and a more physiological uptake when combined with foods (58). This safety is somewhat represented in our results, with only 1/5 “traditional” fortification studies and 2/5 sprinkle studies leading to an increased incidence of diarrhea [Table 4]. Although it appears that, when compared to fortificants, supplements have a higher risk of diarrheal morbidity, it is important to note that the data on the effectiveness of each intervention were not extracted. For example, a study providing low-dose iron fortification may have no effect on morbidity but also no effect on the intended outcome of interest; usually serum ferritin. This limitation of this review makes it difficult to recommend a specific form of intervention.

### Limitations

The search term “iron” was an essential keyword in the search strategy used. It is possible that a select few multiple micronutrient studies would not have included the keyword “iron,” as it may have been an assumed “micronutrient” in the collective whole. This constraint was unavoidable if all iron interventions were to be captured, and a number of reviews already exist that assess the safety of multiple micronutrient interventions (60).

“Conventional vote-counting” (61) was the method used to describe the results of this review. This involves counting the number of trials that showed an adverse effect of the intervention on diarrhea (7/19), those that had a protective effect (0/19) and those that had no effect (12/19). A great deal of literature has been published on vote counting and its inherent flaws, which this study is also fallible to (62, 63). In order to mitigate these effects, this review only “counted” positive associations that were statistically significant at a significance level of *P* < 0.05, whether that be in a specific subgroup or overall. This adaptation provides a more robust overview of relations than older forms of conventional vote-counting that often use a baseline cutoff of *P* < 0.5 (64).

There was significant heterogeneity in how diarrheal outcomes were reported. Proportions, risk ratios, incidence rates, and raw numbers were all variously reported. However, “incidence” definitions varied, with some studies reporting total frequency of diarrheal episodes and others reporting the number of children who ever suffered from diarrhea within a given period. The latter value always gives a number below that of the total study population (*n*), whereas the former could be much higher, as it accounts for children who suffer from multiple discrete episodes of diarrhea throughout the study duration.

## Conclusions

Undoubtedly there is a strong need for effective treatments for iron deficiency. However, a delicate balance between providing iron to host and increasing pathogen growth needs to be maintained, particularly in the gut. Factors such as genetics, gut integrity, diet, hygiene, and inflammation status all contribute to the complex interplay between iron and the gut (65, 66).

We recommend that future iron-intervention studies consider 3 key recommendations. First, diarrhea as defined by the WHO should be recorded as a clearly reported secondary outcome, preferably as a crude number. Second, antibiotic status of individuals enrolled in an iron study should be collected, with those taking antibiotics at baseline excluded. This would help account for possible drug interactions and the possibility of the “red stool effect.” Finally, fecal bacteria should be analyzed where possible to help contribute to the burgeoning field of microbiome research and to further understand the selective effects of iron on specific bacterial species. We hope that these recommendations are modest yet sufficiently achievable to ensure that diarrhea is adequately assessed in iron-intervention studies.

## Supplementary Material

Supplement File

## Notes

Funded by Bill and Melinda Gates Foundation and core funding MC-A760-5QX00 to the International Nutrition Group by the UK Medical Research Council and the UK Department for International Development under the Medical Research Council/Department for International Development Concordat agreement.

Author disclosures: None of the authors report a conflict of interest related to research presented in this article.

Supplemental Tables 1–6 are available from the “Supplementary data” link in the online posting of the article and from the same link in the online table of contents at https://academic.oup.com/cdn/.
