# Supplementary material for: Guts, Germs, and Iron: A Systematic Review on Iron Supplementation, Iron Fortification, and Diarrhea in Children Aged 4–59 Months
Source: Curr Dev Nutr. 2019 Jan 15;3(3):nzz005. doi: 10.1093/cdn/nzz005 (PMC6416531; doi:10.1093/cdn/nzz005)
Supplement: Supplement File [file nzz005_supplement_file.docx]

## Supplemental Table 1: Search Strategy

### Ovid 516 ­Results (31/07/17)

((child OR infant) AND (iron OR ferrous OR ferric or Fe OR FeFol) AND (MNP OR MMN OR Chispitas OR packet OR powder OR fort* OR supplem* OR micronutrient) AND (diarrhoea OR diarrhea OR gastroenteritis OR dysentry)).mp. [mp=ab, ti, ot, bt, hw, id, cc, nm, kf, px, rx, an, ui, sy, tn, dm, mf, dv, kw, fx]

### CENTRAL 161 Results (31/07/17)

ID Search Hits

#1 infant (42349)

#2 child (100724)

#3 Iron (6857)

#4 ferrous (112)

#5 ferric (923)

#6 fefol (7)

#7 fe (4445)

#8 MNP (67)

#9 MMN (270)

#10 Micronutrient (1214)

#11 Chispitas (2)

#12 packet (277)

#13 powder (4778)

#14 fort* (4143)

#15 Supplem* (60925)

#16 diarrhoea (4809)

#17 diarrhea (19046)

#18 Gastroenteritis (1471)

#19 Dysentry (3)

*#20 (#1 OR #2) AND (OR #3-#7) AND (OR #8-#15) AND (OR #16-#19):ti,ab,kw (Word variations have been searched) (161)*

### Web of Science 198 results (31/07/17)

Query: TOPIC: ((child OR infant) AND (iron OR ferrous OR ferric OR Fe OR FeFol) AND (MNP OR MMN OR Chispitas OR packet OR powder OR fort* OR supplem*) AND (diarrhoea OR diarrhea OR gastroenteritis OR dysentry))

## Supplemental Table 2: Excluded Studies with Reasons

| **Study** | **Year** | **Reason for Exclusion** |
| --- | --- | --- |
| Abbeddou et al | 2015 | *Lipid-based supplement* |
| Abrams et al | 2003 | *Age* |
| Adetifa & Okomo | 2009 | *Design-Review* |
| Agustina et al | 2007 | *Treatment* |
| Alarcorn et al | 2004 | *Inadequate Placebo/Control* |
| Amini-Ranjbar et al | 2007 | *Treatment* |
| Anderson et al | 2008 | *Irrelevant Study/Outcome* |
| Arsenault et al | 2009 | *Age* |
| Ashong et al | 2012 | *Design-Protocol* |
| Barness et al | 1978 | *Design* |
| Baumgartner & Barth-Jaeggi | 2015 | *Design-Review* |
| Bégin et al | 2008 | *Irrelevant Study/Outcome* |
| Berger et al | 2013 | *Design* |
| Bhandari et al | 2007 | *Zinc Supplementation* |
| Bhutta | 2007 | *Irrelevant Study/Outcome* |
| Busner et al | 1993 | *Milk/Formula* |
| Chen et al | 2016 | *Milk/Formula* |
| Chhagan et al | 2009 | *Identical cohort: Reported on by Luabeya et al., 2003* |
| Chhagan et al | 2010 | *Identical cohort: Reported on by Luabeya et al., 2003* |
| Christiofides et al | 2005 | *Design* |
| Christofides et al | 2006 | *Inadequate Placebo/Control* |
| Cross et al | 2015 | *Irrelevant Study/Outcome* |
| Das et al | 2017 | *Design-Protocol* |
| de Baros & Cardoso | 2016 | *Design* |
| De Romana et al | 2003 | *Irrelevant Study/Outcome* |
| De-Regil et al | 2011 | *Design* |
| De-Regil et al | 2012 | *Design* |
| De-Regil et al | 2013 | *Design-Review* |
| Dostal et al | 2014 | *Age* |
| Duque et al | 2014 | *Inadequate Placebo/Control* |
| Dutta et al | 2011 | *Treatment* |
| Eneroth et al | 2009 | *Irrelevant Study/Outcome* |
| Feeney et al | 2013 | *Not Accessible* |
| Fei et al | 2014 | *Milk/Formula* |
| Filteau et al | 2013 | *Design-Not RCT* |
| Fischer Walker et al | 2009 | *Identical cohort: Reported on by Baquie et al, 2003* |
| Freycon & Poyau | 1983 | *Irrelevant Study/Outcome* |
| Geltman et al | 2009 | *Inadequate Placebo/Control* |
| Gera & Sachdev | 2002 | *Design-Review* |
| Gera et al | 2003 | *Design-Review* |
| Gessner et al | 2006 | *Irrelevant Study/Outcome* |
| Grant et al | 1972 | *Milk/Formula* |
| Heresi et al | 1987 | *Milk/Formula* |
| Heresi et al | 1995 | *Milk/Formula* |
| Hernandez et al | 2012 | *Design* |
| Hirve et al | 2007 | *Inadequate Placebo/Control* |
| Hossain et al | 2016 | *Irrelevant Study/Outcome* |
| Iannotti et al | 2009 | *Irrelevant Study/Outcome* |
| Iannotti et al | 2014 | *Lipid-based supplement* |
| Iannotti et al | 2010 | *Zinc Supplementation* |
| Imdad et al | 2016 | *Irrelevant Study/Outcome* |
| Imdad et al | 2017 | *Irrelevant Study/Outcome* |
| Irigoyen et al | 1991 | *Milk/Formula* |
| Irlam et al | 2013 | *Design-Review* |
| Irlam et al | 2013 | *Irrelevant Study/Outcome* |
| Iwanczak & Francavailla | 2014 | *Irrelevant Study/Outcome* |
| Juyal et al | 2004 | *Milk/Formula* |
| Krebs et al | 2015 | *Irrelevant Study/Outcome* |
| Lancaster et al | 2008 | *Design-Case Report* |
| Lazzerini | 2014 | *Design-Review* |
| Lazzerini et al | 2013 | *Formulated Food* |
| Lemaire et al | 2011 | *Identical cohort: Reported on by Lemaire et al, 2010* |
| Leme Coutinho et al | 2008 | *Treatment* |
| Li et al | 2000 | *Treatment* |
| Lind et al | 2008 | *Treatment* |
| Long et al | 2007 | *Design* |
| Longfils et al | 2008 | *Irrelevant Study/Outcome* |
| Lopriore et al | 2004 | *Inadequate Placebo/Control* |
| Low et al | 2013 | *Design-Review* |
| Lutter et al | 1988 | *Design-Whole food supplementation* |
| Lutter et al | 1990 | *Formulated Food* |
| Mangani et al | 2014 | *Lipid-based supplement* |
| Manger et al | 2008 | *Irrelevant Study/Outcome* |
| McCormick et al | 2010 | *Milk/Formula* |
| Mda et al | 2010 | *Irrelevant Study/Outcome* |
| Naude et al | 2000 | *Irrelevant Study/Outcome* |
| Ndeezi et al | 2012 | *Irrelevant Study/Outcome* |
| Neuberger et al | 2016 | *Design-Review* |
| Ochoa et al | 2008 | *Milk/Formula* |
| Paganini et al | 2016 | *Design* |
| Palacios et al | 2017 | *Language-Spanish* |
| Peña-Rosas et al | 2014 | *Design-Protocol* |
| Penny et al | 2004 | *Treatment* |
| Pereira et al | 2014 | *Irrelevant Study/Outcome* |
| Petry et al | 2016 | *Design-Review* |
| Rah et al | 2012 | *Design* |
| Ramakrishnan & Goldenberg | 2011 | *Design-Review* |
| Rameshwar et al | 2006 | *Irrelevant Study/Outcome* |
| Reinhart et al | 2012 | *Inadequate Placebo/Control* |
| Roma & Miele | 2015 | *Irrelevant Study/Outcome* |
| Sachdev | 2003 | *Not Accessible* |
| Salam et al | 2013 | *Design-Review* |
| Sazawal et al | 2007 | *Milk/Formula* |
| Sampaio et al | 2004 | *Inadequate Placebo/Control* |
| Scariati et al | 1997 | *Milk/Formula* |
| Schoonees et al | 2013 | *Design-Review* |
| Schwartz et al | 1994 | *Irrelevant Study/Outcome* |
| Self et al | 2012 | *Design-Protocol* |
| Sharieff et al | 2006 | *Design* |
| Sharieff et al | 2006 | *Treatment* |
| Singhal et al | 2000 | *Milk/Formula* |
| Smuts et al | 2005 | *Inadequate Placebo/Control* |
| Solomons | 2002 | *Irrelevant Study/Outcome* |
| Suchdev et al | 2008 | *Design-Marketing/multi-intervention* |
| Szymlek-Gay et al | 2009 | *Milk/Formula* |
| Taneja et al | 2009 | *Zinc Supplementation* |
| Tielsch et al | 2006 | *Age* |
| Tobe-Gai & Mori | 2013 | *Irrelevant Study/Outcome* |
| Van der Kam et al | 2016 | *Treatment* |
| van Stuijvenberg et al | 1999 | *Irrelevant Study/Outcome* |
| Veenemans et al | 2012 | *Zinc Supplementation* |
| Wang et al | 2016 | *Irrelevant Study/Outcome* |
| Warady et al | 2006 | *Irrelevant Study/Outcome* |
| Wasantwisut et al | 2006 | *Irrelevant Study/Outcome* |
| Weippl | 1978 | *Language-German* |
| Wieringa et al | 2010 | *Irrelevant Study/Outcome* |
| Windle et al | 2013 | *Irrelevant Study/Outcome* |
| Wu & Tsai | 2016 | *Irrelevant Study/Outcome* |
| Young et al | 2017 | *Not Accessible* |
| Yurakok et al | 2004 | *Inadequate Placebo/Control* |
| Zavaleta et al | 2011 | *Irrelevant Study/Outcome* |
| Ziegler | 2011 | *Milk/Formula* |
| Zimmerman et al | 2010 | *Irrelevant Study/Outcome* |
| Zlotkin et al | 2001 | *Inadequate Placebo/Control* |

## Supplemental Table 3: Population Characteristics and Study Design

| **Study** | **Comorbidity** | **Type** | **Cluster vs Individual** | **Recruitment Site** | **Case Definition** | **Case Detection** | **Eligibility** | **Exclusion Criteria** |
| --- | --- | --- | --- | --- | --- | --- | --- | --- |
| Abdelrazik et al | None | Supplement | Individual | Hospital | 3 Loose Stools per day, | Reported by mother via written calendar during routine visits to Healthy Baby Clinic | Attend routine healthy baby clinical assessment unit | Congenital anomalies, metabolic disorders, abnormal bloods (excluding anaemia) |
| Baqui et al | None | Supplement | Individual | Community | 3 Loose Stools per day, 1 loose bloody stool | Community Health Worker Weekly Visit | Sampling frame - infants in Matlab Surveillance System Database. | Formula-fed, wasted, physically disabled, neurological disorders, chronic illness, severe anaemia. |
| Barth-Jaeggi et al | None | Fortified Maize Porridge | Individual | Hospital recruitment | Diarrhoea- undefined | Nurse questioning at clinic appointment | Recruitment not detailed | Non-anaemic, no acute or chronic illness. |
| Chang et al | None | Supplement | Individual | Hospital recruitment | 3 Loose Stools in two consecutive days. | Weekly Village health worker visits. | Randomly chosen child from selected local villages (convenience sample) | Wasted, anaemic, chronic illness, active fever. |
| Chen et al(2011) | None | Fortified cereal | Individual | Community  (Nurseries) | 3 Loose Stools per day | Biweekly household interviews by health workers | CRP <10mg/L, consent, no supplement use | Hb level <60/g/L, chronic illness, acute illness |
| Chen et al(2013) | None | Supplement | Individual | Community  (Kindergarten) | 3 Loose Stools per day, | Biweekly caretaker interviews | Random recruitment from eight regional kindergartens | Non-anaemic, no acute or chronic illness, C-reactive P<10mg/L |
| Christofides et al | None | Point of use fortification | Individual | Community  (field) | Diarrhoea- undefined | Biweekly household interviews by health workers | All children within geographical area within age range | Hb <100 g/L |
| Dewey et al | None | Supplement | Individual | Hospital Recruitment | 3 Loose Stools per day | Stool diary by mother | Sweden: Hospital birth, 20km residence. Honduras: Hospital birth | Normal birthweight, term birth, Breastfed, non-anaemic, |
| Giovannini et al | None | Point of use fortification | Individual | Community | Diarrhoea- undefined | Weekly Village health worker visits. | Random sample of 28 villages in the local area. Infants aged 6 months +-7days at time of recruitment. | Severe Anaemia |
| Jaeggi et al | None | Fortified Maize Porridge | Individual | Community | Weekly multiple choice questionnaire | Weekly multiple choice questionnaire, nurse confirmation | Age, Mother >15years old, | Antibiotic use, anaemia, chronic diseases |
| Javaid et al | None | Fortified Cereal | Individual with separate village control | Community | 4 Loose Stools per day | Weekly Village health worker visits. | Cohort from previous population survey. Normal birth weight. | Chronic disease, haemolytic anaemia |
| Lemaire et al | Moderate Acute Malnutrition | Point of use fortification | Individual | Community  (field) | 3 loose stools per day, 1 bloody stool | Every 2 days questionnaire administered by field worker | weight-for-age z score between -2 to -3 | haemoglobin con- centration <70 or >110 g/L, oedema, iron supplementation, congenital abnormalities, iron supplementation |
| Luabeya et al | HIV infected children included | Supplement | Individual | Community  (field) | increased stool frequency (no number), watery stool, blood in stool | Weekly household interviews by health workers with mother | Children recruited from routine hospital clinic visits | Oedema, weight below 60% of median, chronic diarrhoea, concurrent supplementation (excluding vitamin A) |
| Menon et al | None | Fortified wheat soy blend | Cluster | Community  (field) | Diarrhoea- undefined | Morbidity data collected monthly | Users of food distribution points within Haiti | Severe anaemia, no mother, not receiving wheat-soy blend |
| Mitra et al | None | Supplement | Individual | Community  (field) | "three or more liquid stools in a 24-h period, except for breast-fed infants," which relied on mother's report. Dysentery- blood in stool. | House visits on alternate days as well as local physician records. | Census/geographic data of Nanidpara Village | Congenital anomalies, metabolic disorders, critical illness |
| Paganini et al | None | Point of use fortification | Individual | Community (field) | Diarrhoea- undefined | Weekly Village health worker visits. | Age, residence area | Chronic disease, concurrent supplementation, prior antibiotic use |
| Richard et al | None | Supplement | Individual | Community  (field) | 3 Loose Stools per day, 1 loose bloody stool | Twice weekly interviews | No chronic Illness or wasting | None barring eligibility criteria |
| Rosado & Allen | None | Supplement | Individual | Community  (field) | Mother's assessment of 'frequent loose stools' | Twice weekly interviews and written questionnaire by field worker | Age 1.5-3 years | None barring eligibility criteria |
| Soofi et al | None | Point of use fortification | Cluster | Community (field) | 3 Loose Stools per day | Maternal report during regular health worker visits | Age, consent | Congenital abnormalities, birth defects, chronic illnesses |

## Supplemental Table 4: Intervention Types

| **Study** | **Route** | **Dose** | **Frequency** |
| --- | --- | --- | --- |
| Abdelrazik et al | Syrup | Titrated to 1mg/kg equivalence of elemental iron | Daily, before breakfast, stopped during fever or infection. |
| Baqui et al | Capsule | Standardised | Weekly |
| Barth-Jaeggi et al | Fortified maize porridge. | 1g | Daily |
| Chang et al | Dissolvable tablets | Half dose for those <12 months. | Placebo- Daily, Iron- alternate days, Zinc- alternate days, iron+zinc combined daily or alternate days |
| Chen et al(2011) | Powder | 1 Packet | Daily |
| Chen et al(2013) | Capsule | Weight Based | Five days a week excluding kindergarten holidays |
| Christofides et al | Powder | 1 packet | Daily |
| Dewey et al | Syrup | Weight Based | Daily |
| Giovannini et al | Sprinkle | One packet | Daily |
| Jaeggi et al | Fortified maize porridge. | N/A | Daily |
| Javaid et al | Fortified cereal | Recorded intake | Daily |
| Lemaire et al | Powder | 1 packet | Daily |
| Luabeya et al | Tablet | 1 tablet | Daily |
| Menon et al | Sprinkles | 1 packet | Daily |
| Mitra et al | Syrup | 5ml | Daily |
| Paganini et al | Powder | 1 packet | Daily |
| Richard et al | Syrup | 2.5ml | Daily |
| Rosado & Allen | Solution | 20ml | Daily |
| Soofi et al | Sprinkles | 1 packet | Daily |

## Supplemental Table 5: Interventions and Ingredients

| **Study** | **Intervention** | **Placebo** | **Other** |
| --- | --- | --- | --- |
| Abdelrazik et al;  2007 | Multisanostol plus iron 5ml: **43 mg ferrous gluconate**  1200 IU vitamin A,  100 IU vitamin D3,  1 mg thiamin,  mg riboflavin,  0.5 mg pyridoxine,  50 mg vitamin C,  1 mg vitamin E,  5 mg nicotinamide,  2 mg panthenol,  50 mg calcium gluconate and phosphate. | Multisanostol 5ml:  1200 IU vitamin A,  100 IU vitamin D3,  1 mg thiamin,  1 mg riboflavin,  0.5 mg pyridoxine,  50 mg vitamin C,  1 mg vitamin E,  5 mg nicotinamide,  2 mg panthenol,  50 mg calcium gluconate and phosphate |  |
| Baqui et al;  2003 | **20mg ferrous sulphate** | 1mg Riboflavin | MMN:  **20mg ferrous sulphate,**  1mg Riboflavin,  20 mg zinc,  100ug iodine,  1.2 mg copper,  1.6 mg manganese,  30ug selenium,  70 mg vitamin C,  20ug vitamin D,  8 mg vitamin E,  0.8 mg thiamin,  12 mg niacin,  1.2mg pyridoxine,  70ug folic acid,  1.0ug cyanocobalamin,  6 mg pantothenic acid. |
| Barth-Jaeggi et al;  2015 | **2.5mg NaFeEDTA,** Vitamin A 100 μg,  Vitamin D 5 μg,  Copper 0.34 mg, Tocopherol 5 mg,  Iodine 30 μg,  Vitamin K1 30 μg,  Selenium 17 μg,  Thiamine 0.5 mg,  Zinc 2.5 mg,  Riboflavin 0.5 mg, Pyridoxine 0.5 mg,  Folic acid 90 μg, Niacinamide 6 mg,  Vitamin B12 0.9 μg, Vitamin C 60 mg | Vitamin A 100 μg,  Vitamin D 5 μg,  Copper 0.34 mg,  Tocopherol 5 mg,  Iodine 30 μg,  Vitamin K1 30 μg,  Selenium 17 μg,  Thiamine 0.5 mg,  Zinc 2.5 mg,  Riboflavin 0.5 mg,  Pyridoxine 0.5 mg,  Folic acid 90 μg,  Niacinamide 6 mg  Vitamin B12 0.9 μg,  Vitamin C 60 mg |  |
| Chang et al;  2010 | **6.25mg Iron**,  50IU Folate | Identical shape,  no added micronutrients. | 5mg Zinc |
| Chen et al;  2011 | **12mg ferric sodium edentate (NaFeEDTA),**  Vitamin A 500 ug | Vitamin A 500 ug | **12mg ferric sodium edentate (NaFeEDTA),**  Vitamin A 500 ug,  thiamine 0.7 mg ,  riboflavin 0.7 mg, folic acid 0.2 mg,  niacinamide 7 mg,  zinc 12 mg,  calcium 800 mg |
| Chen et al;  2013 | **Ferrous sulphate 1-2mg/kg** | Placebo non-descript | 20,000 IU Vit A  **Ferrous Sulphate** |
| Christofides et al;  2005 | **30 mg ferrous fumarate,**  150 µg folic acid,  50 mg L-ascorbic acid,  200 IU vitamin D3. | Ground purple rice. |  |
| Dewey et al;  2002 | **Ferrous sulphate 1mg/kg,**  sugar,  citric acid,  sodium bisulphate, flavouring. For 5 months. | Placebo | **Ferrous sulphate 1mg/kg,** sugar, citric acid, sodium bisulphate, flavouring. For 3 months. |
| Giovannini et al;  2006 | MMN: **iron II fumarate 12.5mg,** Zn 5mg,  Vitamin C 50 mg,  Vitamin A 300ug Vitamin D3 7.5ug,  Folic acid 150ug,  Potato maltodextrins 1g, | Placebo Potato maltodextrins 1g | FFA:  **Fe (iron II fumarate) 12.5mg,**  Folic acid 150ug Potato maltodextrins 1g |
| Jaeggi et al;  2014 | **12.5mg ferrous fumarate**, Zinc 5mg,  Vitamin A 300mg,  Vitamin C 30mg | Zinc 5mg,  Vitamin A 300mg,  Vitamin C 30mg |  |
| Javaid et al;  1991 | Cerelac cereal,  **ferrous fumarate mean intake=4.1-5.1mg** (high bioavailability) | Cerelac cereal | Cerelac Cereal,  ferric-pyrophospohate mean intake mean intake 4.1mg (low bioavailability) |
| Lemaire et al;  2010 | **12.5 mg ferrous fumarate**,  vitamin A 400 ug,  zinc gluconate 5 mg, vitamin C 30 mg,  folic acid, 0.15mg, maltodextrin. | Maltodextran |  |
| Luabeya et al;  2007 | **10 mg ferrous fumarate,** vitamin A 1250 IU,  zinc 10mg,  0.5 mg B1/B2/B6,  0.9 mg vitamin B12,  35 mg vitamin C,  5 mg vitamin D,  6 mg vitamin E,  10 mg vitamin K,  0.6 mg copper as cupric gluconate,  150 mg folate;  50 mg iodine,  6 mg niacin. | Vitamin A 1250 IU | Vitamin A 1250 IU,  zinc 10mg, |
| Menon et al;  2007 | **12.5 mg iron (non-descript)**,  5mg zinc, 400ug  vitamin A, 160ug  folic acid, 30mg  vitamin C,  wheat soy blend | Wheat soy blend |  |
| Mitra et al;  1997 | 400 RE Vitamin A,  10ug Vitamin D,  50mg Vit C,  **Ferrous Gluconate 125mg** | 400 RE Vitamin A,  10ug Vitamin D,  50mg Vit C, |  |
| Paganini et al;  2017 | **ferrous fumarate 2.5mg,**  **NaFEDTA 2.5mg,**  Maltodextrin 11g,  Vitamin A 400 μg,  Vitamin D 5μg,  Tocopherolequivalents 5 mg,  Thiamine 0.5 mg,  Riboflavin 0.5 mg,  Vitamin B6 0.5 mg,  Folic acid 90 μg,  Niacin 6mg,  Vitamin B12 0.9 μg,  Vitamin C 30 mg,  Copper 0.56 mg,  Iodine 90 μg,  Selenium 17 μg,  Zinc 4.1 mg,  Phytase 190 FTU | Maltodextrin 11g,  Vitamin A 400 μg,  Vitamin D 5μg,  Tocopherolequivalents 5 mg,  Thiamine 0.5 mg,  Riboflavin 0.5 mg,  Vitamin B6 0.5 mg,  Folic acid 90 μg,  Niacin 6mg,  Vitamin B12 0.9 μg,  Vitamin C 30 mg,  Copper 0.56 mg,  Iodine 90 μg,  Selenium 17 μg,  Zinc 4.1 mg,  Phytase 190 FTU | **ferrous fumarate 2.5mg, NaFEDTA 2.5mg,**  7.5g Galacto-oligosaccharides,  Maltodextrin 11g,  Vitamin A 400 μg,  Vitamin D 5μg,  Tocopherolequivalents 5 mg,  Thiamine 0.5 mg,  Riboflavin 0.5 mg,  Vitamin B6 0.5 mg,  Folic acid 90 μg,  Niacin 6mg,  Vitamin B12 0.9 μg,  Vitamin C 30 mg,  Copper 0.56 mg,  Iodine 90 μg,  Selenium 17 μg,  Zinc 4.1 mg,  Phytase 190 FTU |
| Richard et al;  2006 | **15mg iron sulphate** | Placebo | **15mg iron sulphate,** 20mg zinc |
| Rosado & Allen;  1997 | **20mg ferrous sulphate**  Sugar,  water,  citric acid,  artificial flavour | Sugar,  water,  citric acid,  artificial flavour | **20mg ferrous sulphate,** 20mg Zinc Sugar, water, citric acid, artificial flavour |
| Soofi et al;  2013 | **12.5mg**  **ferrous fumarate,**  50mg Vitamin C,  300ug Vitamin,  5ug Vitamin D,  50ug Folate | Control group/no intervention | **12.5mg ferrous fumarate,**  50mg Vitamin C,  300ug Vitamin,  5ug Vitamin D,  150ug Folate,  10mg Zinc |

## Supplemental Table 6: Risk of Bias Supporting Statements

| **Study** | **Random Sequence Generation**  **(Selection Bias)** | **Allocation Concealment**  **(Selection Bias)** | **Blinding**  **(Performance)** | **Blinding**  **(Detection)** | **Incomplete Data**  **(Attrition Bias)** | **Selective**  **(Reporting Bias)** | **Other Bias** | **Comments** |
| --- | --- | --- | --- | --- | --- | --- | --- | --- |
| Abdelrazik et al | Randomised control trial, unclear method | Randomised control trial, unclear method | Unclear risk | None Described | Attrition reported, specific numbers in each group not reported | Unlikely, almost all domains, subgroups and multiple outcomes reported with specific breakdowns of each. | Low Risk |  |
| Baqui et al | Randomised allocation, unclear method | Randomised allocation, unclear method | "double blind" capsules made non-descript | None Described | Thorough attrition data given with reasons for exclusion including flow diagram | All data reported on stated characteristics, other outcomes 'reported elsewhere' | Low Risk |  |
| Barth-Jaeggi et al | Computer generated allocation. | Computer generated allocation. | "Triangle test" conducted to ensure acceptability and indistinguishability between interventions | Double Blinded, clinicians only know of enrolment | Accounted for in detail, an error made during MNP production led to invalid results which are excluded. | Morbidity reporting methods are unconventional, reported numbers are given as average days of morbidity, however percentage data also presented | Low Risk | Very Thorough |
| Chang et al | Computer generated block randomisation | Computer generated block randomisation | Manufacturer blinded packaging. | Allocation revealed at the end of the trial via sealed envelopes | Protocol deviations, dropouts and reasons accounted for | Unlikely, unaccounted data described and mentioned in analysis. | Low Risk | Very Thorough |
| Chen et al (2011) | Randomisation by external investigator, possibly inadequate | Randomisation by external investigator, possibly inadequate | Participants physically separated, powders indistinguishable in taste and colour | Children, teachers and assessors not aware of allocation until after the study. | 56 Children dropped out, reasons for attrition detailed | Unclear |  |  |
| Chen et al (2013) | Excel-based randomisation, stratified to ensure equal spread of base-line characteristics. | Excel-based randomisation, stratified to ensure equal spread of base-line characteristics. | "Children unaware of intervention assignment" | "Analysts, assessors and workers…unaware." | Attrition reported, high dropout rate, though reasons for attrition unaccounted | Lots of outcomes reported in a number of ways but results difficult to untangle. A clear narrative to promote the combination of Vitamin A + Iron is seen. | Low Risk |  |
| Christofides et al | Using poker chips pulled by hand from a bag. | Using poker chips pulled by hand from a bag. | Identical taste plain sachets | Not discussed | 40 children dropped out, reasons given, not in detail | Unlikely, almost all domains, subgroups and multiple outcomes reported with specific breakdowns of each. |  |  |
| Dewey et al | Stratified Randomisation | Stratified Randomisation | Placebo solution with similar colour and taste | "Double blinded" details unclear | Dropout numbers given, raw numbers not detailed- "no difference" between dropouts and non-drop outs | Complicated trial, results use a combination of adjustments- may be liable to P-fishing. | Low risk |  |
| Giovannini et al | Randomisation' non-descript. | Randomisation' non-descript. | Lot and internal batch number on packet only. | Sealed envelopes, personnel unaware of allocation until analysis. | CONSORT flow diagram accounting for drop outs and reasoning included, | Diarrhoea outcome was not reported in detail as it was a secondary outcome, 12-month diarrhoea incidence not provided | Low Risk | Poor quality for the outcome of diarrhoea |
| Jaeggi et al | Randomly assigned' | Randomly assigned' | Triangle taste tests and identical packaging | Non-described | Infants excluded for various reasons including antibiotic use | Morbidity data for low iron subset of fortification not reported |  |  |
| Javaid et al | Randomisation non-descript | Randomisation non-descript | No allocation concealment described | No allocation concealment described | Older study, no CONSORT diagram but drop outs and exclusions accounted for | Contrary to most clinical guidelines. Diarrhoea was reported as four incidents per day, morbidity data was combined for the two iron containing groups. There is no logical reason why this should be the case | High Risk, unusual reporting methods | High risk of uncontrolled confounding, control group differs greatly from the intervention. |
| Lemaire et al | Microsoft Excel block randomisation | Microsoft Excel block randomisation | Identical taste, packaging, colour | All participants and researchers blinded until end of trial | Full robust CONSORT diagram with reasons for attrition | Primary outcome was an unorthodox composite score combining dysentery, diarrhoea and LRTI. Specific numbers for each individual outcome not even mentioned. Supplemental data had to be sourced in order to assess effects. |  |  |
| Luabeya et al | Computer randomised block allocation stratified by HIV status | Computer randomised block allocation stratified by HIV status | Tablets plain packaged and numbered, similair colour, size and taste | "Investigators, study staff and participants were blind to the treatment assignments" | Highly detailed patient flowchart provided with reasons for attrition. | Unlikely, almost all domains, subgroups and multiple outcomes reported with specific breakdowns of each. | Weak case definition | weak case definition |
| Menon et al | Randomisation of food distribution points- method not detailed | Randomisation of food distribution points- method not detailed | Intervention received sprinkles, placebo group received food only | Blinding not applicable | Full robust CONSORT diagram with reasons for attrition | Specific numbers of diarrhoea incidence in each group not provided |  |  |
| Mitra et al | Two stage randomisation of four clusters leading to equal numbers of each group per cluster. | Two stage randomisation of four clusters leading to equal numbers of each group per cluster. | Syrup in identical bottles, smell and taste. | Group allocation revealed after analysis | All attrition accounted for. 'No indication of differential attrition' | Unlikely, unaccounted data described and mentioned in analysis. | Low Risk | High quality study. |
| Paganini et al | Computer generated allocation. | Computer generated allocation. | Discreet coded sachets | Participants and Investigators masked | Full CONSORT flow diagram, with reasons for drop out | No case definition for diarrhoea, incidence numbers for diarrhoea in each group not provided. | Low Risk |  |
| Richard et al | Algorithm SAS 6 used to randomise groups | Algorithm SAS 6 used to randomise groups | Supplements similar in taste and colour | Personnel and analysts masked until seminal analysis stage | Full CONSORT flow diagram, however no breakdown for age specific strata which is needed for this review. | Unlikely, however full data for each stratum is not reported | Low Risk |  |
| Rosado & Allen | Stratified Randomisation | Stratified Randomisation | Supplements identical in taste and colour | Double blinded' details unclear | 25 children dropped out, due to family situation. | Unlikely, almost all domains, subgroups and multiple outcomes reported with specific breakdowns of each. | Weak case definition. |  |
| Soofi et al | Computer generated cluster randomisation by rural/urban strata | Computer generated cluster randomisation by rural/urban strata | Families blinded to zinc content. However, no placebo powder used in control group. | Families, investigators and staff blinded to zinc content but were aware of use of sprinkles. | Full diagram detailing attrition provided. | Supplementary material models 'missingness' for incomplete data. |  |  |
